# Preconceptional and gestational weight trajectories and risk of delivering a small-for-gestational-age baby in rural Gambia^1^,^2^

**DOI:** 10.3945/ajcn.116.144196

**Published:** 2017-05-10

**Authors:** William Johnson, Seham AA Elmrayed, Fatou Sosseh, Andrew M Prentice, Sophie E Moore

**Affiliations:** 3 School of Sport, Exercise and Health Sciences, Loughborough University, Loughborough, United Kingdom;; 4 Medical Research Council (MRC) Elsie Widdowson Laboratory, Cambridge, United Kingdom;; 5 Nutrition Theme, MRC Unit The Gambia, Banjul, Gambia;; 6 MRC International Nutrition Group, London School of Hygiene & Tropical Medicine, London, United Kingdom; and; 7 Division of Women's Health, King's College London, London, United Kingdom

**Keywords:** age-related trajectories, gestational weight gain, maternal nutrition, prepregnancy weight, small for gestational age

## Abstract

**Background:** Maternal nutritional status is a key determinant of small for gestational age (SGA), but some knowledge gaps remain, particularly regarding the role of the energy balance entering pregnancy.

**Objective:** We investigated how preconceptional and gestational weight trajectories (summarized by individual-level traits) are associated with SGA risk in rural Gambia.

**Design:** The sample comprised 670 women in a trial with serial weight data (7310 observations) that were available before and during pregnancy. Individual trajectories from 6 mo before conception to 30 wk of gestation were produced with the use of multilevel modeling. Summary traits were expressed as weight *z* scores [weight *z* score at 3 mo preconception (*z*wt_−3 mo_), weight *z* score at conception, weight *z* score at 3 mo postconception, weight *z* score at 7 mo postconception (*z*wt_+7 mo_), and conditional measures that represented the change from the preceding time] and were related to SGA risk with the use of Poisson regression with confounder adjustment; linear splines were used to account for nonlinearity.

**Results:** Maternal weight at each time point had a consistent nonlinear relation with SGA risk. For example, the *z*wt_−3 mo_ estimate was stronger in women with values ≤0.5 (RR: 0.736; 95% CI: 0.594, 0.910) than in women with values >0.5 (RR: 0.920; 95% CI: 0.682, 1.241). The former group had the highest observed SGA prevalence. Focusing on weight change, only conditional *z*wt_+7 mo_ was associated with SGA and only in women with values >−0.5 (RR: 0.579; 95% CI: 0.463, 0.724).

**Conclusions:** Protection against delivering an SGA neonate offered by greater preconceptional or gestational weight may be most pronounced in more undernourished and vulnerable women. Independent of this possibility, greater second- and third-trimester weight gain beyond a threshold may be protective. This trial was registered at http://www.isrctn.com/ as ISRCTN49285450.

## INTRODUCTION

Approximately 32.4 million babies were born small for gestational age (SGA)^6^ in 2010, accounting for 27% of births in low- and middle-income countries (LMICs) (1). These babies have an increased risk of stunting, neurocognitive impairment, neonatal and infant mortality, and other outcomes including chronic disease in adulthood (2–8). As part of the 2013 Maternal and Child Nutrition series in *The* *Lancet*, Black et al. (9) estimated that, in total, SGA accounts for 12% of deaths in children aged <5 y.

Maternal nutrition is a key determinant of fetal growth and, therefore, an important target for SGA prevention (10). Part of the relevant literature comprises studies in which maternal weight has been used as an indicator of nutritional status with the aim of providing information about which women are most at risk of delivering an SGA baby and at what time points an intervention might be most effective. The majority of studies have reported positive relations of prepregnancy weight or gestational weight gain with birth weight (11–24), thereby suggesting a need to improve nutritional status before conception as well as during pregnancy.

Despite growing evidence that nutritional status in the preconceptional period can have an effect on SGA risk, there remain some limitations and knowledge gaps. Prepregnancy weight is normally assessed retrospectively and is a single static measurement that does not capture the extent to which a women may be losing or gaining weight (i.e., energy balance) as she enters pregnancy (25). Similarly, gestational weight gain is normally computed as the change in weight between the beginning and end of pregnancy and, as such, cannot be used to reveal the specific temporal pattern of weight change across pregnancy that incurs greatest risk (25). The aim of the present study was to conduct a secondary analysis in a prenatal trial with maternal anthropometry measured serially before and during pregnancy to investigate how preconceptional and gestational weight trajectories (summarized by individual-level traits) are associated with SGA risk. Distinct seasonality in rural Gambia (dry/harvest in November to May; rainy/hungry in June to October) makes rural Gambia an ideal study setting because it produces a diverse range of body weights and energy balances in all adults, including pregnant women, which are closely linked to nutritional factors and physical activity levels (26–30).

## METHODS

### Study

The ENID (Early Nutrition and Immune Development) trial is a randomized trial to assess whether nutritional supplementation to pregnant women (from <20 wk of gestation to term) and their infants (from 6 to 12 mo of age) can enhance immune development (31). Within the ENID trial, pregnancies were identified through monthly surveillance in all eligible nonpregnant women of reproductive age (18–45 y) in the West Kiang region of The Gambia; the date of the last menstrual period was assessed and a urine test was conducted if a menstrual period was missed. Women who were confirmed via ultrasound as being between 10 and 20 wk pregnant at a clinic booking visit were randomly assigned to one of the following 4 arms: *1*) iron and folic acid, *2*) multiple micronutrients (MMNs), *3*) protein energy (PE), and *4*) PE plus MMNs and were supplemented from entry into the trial until delivery. The first women started to receive supplementation in January 2010, and the final infant was born in February 2014.

### Ethics

The trial was approved by the joint Gambia Government/MRC Unit, The Gambia Ethics Committee (project SCC1126v2). Written informed consent was obtained from all women before enrollment in the trial. The trial observed Good Clinical Practice Standards and the current version of the Helsinki Declaration. The trial was registered at http://www.isrctn.com/ as ISRCTN49285450.

### Sample

Three samples were selected from the total ENID cohort (*n* = 875) (**Supplemental Figure 1**). The first sample comprised participants with data that were necessary to model maternal weight trajectories (*n* = 670), and the second sample comprised participants with data that were necessary to relate trajectory traits to SGA risk (*n* = 519); defining characteristics were not different between included and excluded participants (data not shown). The third sample comprised a subsample of the second, which was selected on the basis of maternal preconception weight, and was used in a subset analysis (see Associations of maternal weight-trajectory traits with SGA) (*n* = 400).

### Measurements

This article uses data from the monthly surveillance visits, clinic visits at booking and 20 and 30 wk of gestation, and a home visit that was performed within 72 h of birth.

At the first clinic visit (booking), gestational age was ascertained on the basis of fetal biometry with the use of a Siemens ACUSON Antares Ultrasound Imaging System [CH6-2 (5.71 MHz) transducer; Siemens Medical Solutions USA Inc.].

Maternal weight was measured at the monthly surveillance visits, and maternal weight and height were measured at the clinic visits, with the use of standard techniques and equipment [Tanita DH305 scales (Tanita Corp.) and Leicester height measure (Seca 214; Seca)]. The maternal date of birth and, thus, age were ascertained from the West Kiang Demographic Surveillance System (32). Maternal parity was computed with the use of questionnaire data that were collected at booking as the number of deliveries (i.e., alive children, dead children, and still births) with the exclusion of abortions.

Birth weight, length, and head circumference were measured in the infant’s home by the study midwife and within 72 h of delivery. Weight was measured with the use of digital infant scales (Seca mobile digital baby scale 334; Seca) with the infant in minimal clothing and to the nearest 10 g. Length was measured on a portable infant rollameter (Rollameter 100; Harlow Healthcare) to the nearest 0.1 cm. Head circumference was measured with the use of standard circumference tapes (Seca). All measures were made with the use of standard protocols, and the equipment was regularly validated.

### Statistics

A 2-stage approach was applied, whereby we modeled trajectories before relating summary traits to SGA risk.

### Maternal weight trajectories

The maternal weight data from the monthly surveillance visits and clinic visits at booking and 20 and 30 wk of gestation were pooled, the time scale was expressed as decimal years from conception, and the limited data that occurred before 6 mo preconception or after 9 mo postconception were dropped. This left 7310 observations with a mean of 10.9 observations/participant (range: 4–15 observations/participant) over a mean of 0.98 y (range: 0.44–1.19 y). All women had ≥1 preconception observation and 2 postconception observations.

Individual trajectories were modeled in a multilevel regression framework (measurement occasion at level 1; individuals at level 2) (33, 34). The shape of the trajectory was specified as a restricted cubic spline with 5 knots at −0.45, −0.25, 0, 0.15, 0.25, and 0.3 y. The constant and spline terms had random effects at level 2 with an unstructured variance-covariance matrix. The date or season of measurement was expressed as a level-1 variable ranging from 0 to 1 (i.e., 1 January to 31 December) and was incorporated into the model with Fourier terms and their interactions with spline terms (35, 36). Finally, maternal age, which is a key determinant of weight, was included as a level-1 variable.

Fit was assessed via diagnostic plots of the level-1 residuals. To show the relation of seasonality with maternal weight, the sample-average trajectory from 6 mo preconception to 30 wk of gestation was plotted for the following 2 circumstances: *1*) conceived at the end of the dry/harvest season and *2*) conceived at the end of the rainy/hungry season. The individual trajectories were summarized by estimated weight at *1*) 3 mo preconception, *2*) conception, *3*) 3 mo postconception (i.e., approximately the end of the first trimester), and *4*) 7 mo postconception (i.e., approximately the target time of the last clinic visit). Weight at 6 mo preconception was not estimated because it was unlikely to provide any further information (in terms of SGA risk) over that obtained with the use of weight at 3 mo preconception. A correlation matrix of the 4 traits and additional traits capturing weight changes was produced. Values (of the same traits) of the sample-average trajectory, according to the month of conception, were also estimated.

### Associations of maternal weight-trajectory traits with SGA

Poisson regression models with robust error variance (37) were used to estimate the RRs of SGA (relative to the appropriate for gestational age) associated with each maternal weight trait, expressed as an internal *z* score [e.g., weight *z* score at conception (*z*wt_0 mo_)]. In addition, a model that incorporated conditional measures (e.g., conditional *z*wt_0 mo_), which were computed as the standardized residuals from regressing weight at one time point on weight at all previous time points, was built to investigate the associations of weight changes between consecutive time points with SGA (38, 39). SGA was defined according to a birth weight for gestational age <10th percentile of the INTERGROWTH-21st standard (40), and appropriate-for-gestational-age was defined as values ≥10th or ≤90th percentiles. Adjustment was made for sex (female compared with male), parity (1–3 or 4–12 compared with 0), maternal age (decimal years), and height (centimeters) at booking, intervention (MMNs, PE, or MMNs plus PE compared with iron and folic acid), and season of birth (first 4 sets of Fourier terms). Exploratory work was used to determine the appropriate number of Fourier terms; **Supplemental Figure 2** shows the association of the season of birth (and, thus, the approximate season of conception) with SGA risk that was captured by the first 4 sets of Fourier terms.

General linear models with infant weight-, length-, and head-circumference-for-gestational-age *z* score outcomes according to the INTERGROWTH-21st standards (40) were also developed. The same adjustments were made as in the SGA models except that the season of birth was included as a binary variable [June to October (rainy/hungry) compared with November to May (dry/harvest)] because the pattern of seasonality was less clear (for the continuous outcomes compared with SGA).

Before building the Poisson and general linear regression models previously outlined, the shape of the association between each maternal weight trait and each outcome was explored with the use of restricted cubic splines. Any nonlinearity was subsequently approximated with the use of linear splines. For example, as shown in **Supplemental Figure 3**, the association of the weight *z* score at 3 mo preconception (*z*wt_−3 mo_) with SGA risk was clearly nonlinear (Supplemental Figure 3A) with an inflection point at 0.5 *z* scores (indicated by the vertical gray line). As such, in the Poisson regression models, *z*wt_−3 mo_ was included with the use of 2 linear spline terms, the first of which explained the association of *z*wt_−3 mo_ with SGA risk if *z*wt_−3 mo_ was ≤0.5 and the second of which explained the association of *z*wt_−3 mo_ with SGA risk if *z*wt_−3 mo_ was >0.5. Supplemental Figure 3B shows an approximately linear association, and in this scenario, the maternal weight trait (i.e., conditional *z*wt_0 mo_) would have been entered into the Poisson regression model as a normal, single term.

To understand the extent to which preconceptional weight was associated with SGA independently of weight during pregnancy, a path model was applied to a subset of participants with *z*wt_−3 mo_ ≤0.5 in whom we showed a protective association of *z*wt_−3 mo_ with SGA risk. This approach quantified mediation, whereas the previously described regression models answer different questions (e.g., about how maternal weight change, captured by conditional measures, is related to SGA independently of weight at previous time points). Before building the path model, maternal weight *z* scores were recalculated with the use of only data on the subset. Paths between maternal weight variables were estimated as general linear regression coefficients, and paths to SGA were estimated as Poisson regression coefficients. Adjustment was made for the season of birth, sex, parity, maternal age at booking, maternal height at booking, and intervention (as in the Poisson regression models). Total, total indirect, and direct effects of each maternal weight trait on SGA were computed as RRs with robust error variances.

All procedures were performed in Stata 14 software (StataCorp LP) with the exception that the path model was performed in MPlus 7.4 software (41). The Stata command runmlwin (StataCorp LP) was used for the multilevel model (42).

## RESULTS

The mean birth-weight-for-gestational-age *z* score was −0.82, indicating that our sample was lighter than the 25th percentile of the INTERGROWTH-21st standard, and 33% of the infants were SGA (**Table 1**).

**TABLE 1:** Description of study sample of 670 Gambian women and infants^1^

	Value
Booking visit	
Maternal age, y	30.3 (25.1, 35.1)^2^
Maternal weight, kg (*n* = 1 missing)	55.6 ± 9.5^3^
Maternal height, cm (*n* = 2 missing)	162.0 ± 5.9
Maternal BMI, kg/m^2^ (*n* = 3 missing)	20.5 (19.0, 22.5)
Thinness (<18.5), *n* (%)	122 (18.3)
Normal weight (18.5–24.9), *n* (%)	477 (71.5)
Overweight or obese (≥25), *n* (%)	68 (10.2)
Parity (*n* = 12 missing), *n* (%)	
0	51 (7.8)
1–3	204 (31.0)
4–12	403 (61.2)
Intervention arm, *n* (%)	
FeFol	175 (26.1)
MMNs	170 (25.4)
PE	157 (23.4)
PE + MMNs	168 (25.1)
Birth visit	
Season of measurement, *n* (%)	
November to May (dry/harvest)	430 (64.2)
June to October (rainy/hungry)	240 (35.8)
Sex, *n* (%)	
M	344 (51.3)
F	326 (48.7)
Gestational age, wk	40.3 (39.3, 41.2)
Preterm (<37 0/7 wk of gestation), *n* (%)	14 (2.1)
Postterm (>41 6/7 wk of gestation), *n* (%)	69 (10.3)
* z* score^4^^,^^5^	
Weight-for-gestational-age (*n* = 127 missing)	−0.82 ± 0.95
Length-for-gestational-age (*n* = 107 missing)	0.00 ± 0.12
Head-circumference-for-gestational-age (*n* = 106 missing)	−0.68 ± 1.16
LBW (<2.5 kg), *n* (%)	54 (9.6)
Macrosomic (≥4.0 kg), *n* (%)	2 (0.4)
SGA (<10th percentile),^4^ *n* (%)	179 (33.0)
LGA (>90th percentile),^4^ *n* (%)	15 (2.8)

1 FeFol, iron and folic acid; LBW, low birth weight; LGA, large for gestational age; MMN, multiple micronutrient; PE, protein energy; SGA, small for gestational age.

2 Median, IQR in parentheses (all such values).

3 Mean ± SD (all such values).

4 Values were based on INTERGROWTH-21st birth-size-for-gestational-age standards.

5 Included infants (*n* = 19 for weight and *n* = 20 for length and head circumference) whose gestational age at birth was outside of the range covered by the INTERGROWTH-21st birth-size-for-gestational-age standards (i.e. >42 6/7 wk of gestation).

### Maternal weight trajectories

**FIGURE 1:**
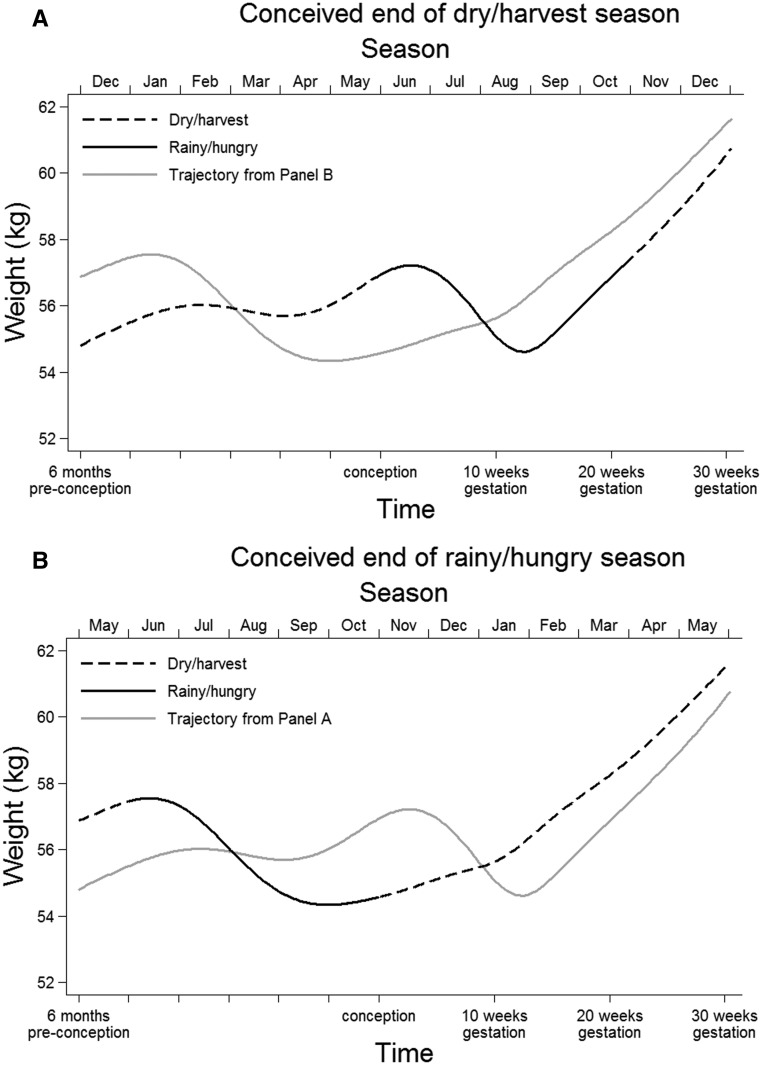
Sample average-weight trajectory from 6 mo preconception to 30 wk gestation in 670 Gambian women according to the season of conception as estimated from the multilevel model reported in** Supplemental Table 1**.

The relation of seasonality with the sample-average maternal weight trajectory is illustrated in **Figure 1**; estimates and a diagnostic plot of the underlying multilevel model are shown in Supplemental Table 1 and **Supplemental Figure 4**. As shown in Figure 1A, mothers who conceived at the end of the dry/harvest season experienced the rainy/hungry season early in pregnancy and, on average, lost ∼2 kg in the first trimester. Conversely, mothers who conceived at the end of the rainy/hungry season weighed ∼3 kg less at conception but showed weight gain across pregnancy and were marginally heavier by 30 wk of gestation (Figure 1B). As shown in **Supplemental Table 2**, values of the sample-average trajectory according to the month of conception (January through December) further showed how seasonality was associated with a diverse range of maternal weight-change patterns.

Estimated traits in the individual trajectories, which were used in subsequent analyses, are summarized in **Supplemental Table 3**. Briefly, the mean weight at conception was 55.5 kg, and the mean weight gain between conception and 7 mo of gestation was 5.5 kg, which fell between the 10th and 25th percentiles of the INTERGROWTH-21st gestational weight-gain standard (43).

### Associations of maternal weight-trajectory traits with SGA

In unadjusted and adjusted Poisson regression models, a higher *z*wt_−3 mo_, *z*wt_0 mo_, *z*wt_+3 mo_, and *z*wt_+7 mo_ were each associated with lower risk of SGA but only in women with values ≤0.5 (**Supplemental Table 4**). Nonlinearity was also observed in the models that investigated the starting weight at 3 mo preconception and the subsequent weight change (**Table 2**). A 1-unit increase in *z*wt_−3 mo_ was associated with ∼26% (RR: 0.736; 95% CI: 0.594, 0.910) reduced risk of SGA in women with values ≤0.5 but only 8% (RR: 0.920; 95% CI: 0.682, 1.241) reduced risk in women with values >0.5. The former group with values ≤0.5 had the highest observed SGA rates. Subsequently, a 1-unit increase in the conditional *z*wt_+7 mo_ was not related to SGA risk in women with values ≤−0.5 (RR: 1.023; 95% CI: 0.763, 1.371) but was associated with ∼42% (RR: 0.579; 95% CI: 0.463, 0.724) reduced risk in women with values >−0.5. This threshold of −0.5 approximated a change of 4.5 kg in maternal weight between 3 and 7 mo of gestation. Also, note that the preconceptional weight change that was captured by the conditional *z*wt_0 mo_ was not associated with SGA risk (RR: 0.928; 95% CI: 0.783, 1.100).

**TABLE 2:** RRs of SGA according to conditional maternal weight measures that were estimated with the use of a single Poisson regression model with robust error variance in 519 Gambian women and infants^1^

		Unadjusted model		Adjusted model	
Maternal or conditional weight *z* score	SGA prevalence, %	RR (95% CI)	*P*	RR (95% CI)	*P*
*z*wt_−3 mo_ (mean ± SD: 54.5 ± 9.1 kg)					
If ≤0.5 *z* scores^2^	36.5	0.749 (0.630, 0.892)	0.001	0.736 (0.594, 0.910)	0.005
If >0.5 *z* scores^2^	24.4	0.936 (0.704, 1.244)	0.649	0.920 (0.682, 1.241)	0.585
Conditional *z*wt_0 mo_ (SD: 1.9 kg)^3^	33.7	0.958 (0.846, 1.083)	0.491	0.928 (0.783, 1.100)	0.390
Conditional *z*wt_+3 mo_ (SD: 2.5 kg)					
If ≤−0.5 *z* scores^2^	43.3	0.871 (0.657, 1.156)	0.340	0.899 (0.652, 1.241)	0.517
If >−0.5 *z* scores^2^	29.8	0.804 (0.646, 1.001)	0.051	0.876 (0.692, 1.109)	0.270
Conditional *z*wt_+7 mo_ (SD: 2.1 kg)					
If ≤−0.5 *z* scores^2^	40.4	1.094 (0.801, 1.493)	0.573	1.023 (0.763, 1.371)	0.882
If >−0.5 *z* scores^2^	30.9	0.612 (0.491, 0.764)	<0.001	0.579 (0.463, 0.724)	<0.001

1 SGA was defined according to a birth-weight-for-gestational-age <10th percentile of the INTERGROWTH-21st standard. Maternal weight *z* scores were calculated internally (i.e., observation − mean/SD) at each time point with the use of the individual weights (kilograms) that were estimated from the multilevel model shown in Supplemental Table 1. Conditional *z* scores were computed as the standardized residuals from regressing weight at one time point on weight at all previous time points with the use of the individual weights (kilograms) that were estimated from the multilevel model shown in Supplemental Table 1; these variables represent the change from the previous time point independent of all previous weights and regression to the mean. The adjusted model was adjusted for the season of birth (first 4 sets of Fourier terms), sex (female compared with male), parity (1–3 or 4–12 compared with 0), maternal age (decimal years) and height (centimeters) at booking, and intervention (multiple micronutrients, protein energy, or multiple micronutrients plus protein energy compared with iron and folic acid). SGA, small for gestational age; *z*wt_0 mo_, weight *z* score at conception; *z*wt_−3 mo_, weight *z* score at 3 mo preconception; *z*wt_+3 mo_, weight *z* score at 3 mo postconception; *z*wt_+7 mo_, weight *z* score at 7 mo postconception.

2 Shape of the association between each maternal or conditional weight *z-*score variable and SGA risk was investigated with the use of restricted cubic splines, and nonlinearity was approximated in the models shown in the table with the use of linear splines.

3 There was no evidence that the association of conditional *z*wt_0 mo_ with SGA risk was nonlinear, which is why only one estimate is presented for this exposure.

**FIGURE 2:**
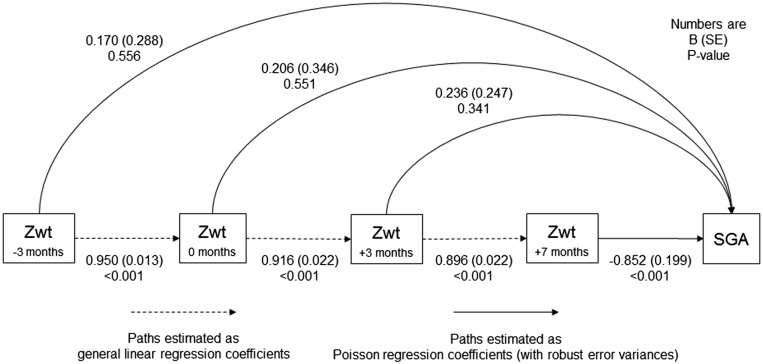
Diagram depicting paths from maternal weight at different time points to SGA, estimated by using a path model applied to a subset of 400 Gambian women and infants. SGA was defined according to a birth weight for gestational age <10th percentile of the INTERGROWTH-21st standard. The model was applied to a subset of participants with a *z*wt_−3 mo_ ≤0.5 *z* scores in whom there was a protective association of a *z*wt_−3 mo_ with SGA risk (Table 2)_._ The model was adjusted for season of birth, sex, parity, maternal age at booking, maternal height at booking, and intervention. With the use of only data on the subset of participants, maternal weight *z* scores were recalculated internally (i.e., observation − mean ÷ SD) at each time point with the use of the individual weights (kilograms) that were estimated from the multilevel model shown in Supplemental Table 1. SGA, small for gestational age; Zwt, weight *z* score.

A diagram depicting the model that was used to estimate the paths between maternal weight at each time point and SGA (in the subset of participants in whom we showed a protective association of the *z*wt_−3 mo_ with SGA risk) is shown in **Figure 2**. The *z*wt_0 mo_ and *z*wt_+3 mo_ had total indirect effects on SGA risk [RR: 0.617 (95% CI: 0.436, 0.872) and 0.466 (0.327, 0.664), respectively] operating through subsequent weight, but only the *z*wt_+7 mo_ had a direct effect (RR: 0.427; 95% CI: 0.289, 0.630) (**Table 3**).

**TABLE 3:** Total, total indirect, and direct paths from maternal weight at different time points to SGA that were estimated with the use of a single-path model that was applied to a subset of 400 Gambian women and infants^1^

	Adjusted model	
Maternal weight *z* score^2^	RR (95% CI)	*P*
*z*wt_−3 mo_ (50.8 ± 5.1 kg)^3^		
Total effect	0.910 (0.794, 1.044)	0.181
Total indirect effect	0.769 (0.446, 1.324)	0.343
Direct effect	1.185 (0.673, 2.085)	0.556
*z*wt_0 mo_ (51.2 ± 5.3 kg)		
Total effect	0.758 (0.427, 1.344)	0.343
Total indirect effect	0.617 (0.436, 0.872)	0.006
Direct effect	1.229 (0.624, 2.421)	0.551
*z*wt_+3 mo_ (51.3 ± 5.3 kg)		
Total effect	0.590 (0.404, 0.862)	0.006
Total indirect effect	0.466 (0.327, 0.664)	<0.001
Direct effect	1.266 (0.780, 2.056)	0.341
*z*wt_+7 mo_ (57.2 ± 5.4 kg)		
Direct effect	0.427 (0.289, 0.630)	<0.001

1 Direct path or effect is the part of the association between a maternal weight trait and SGA that does not operate via any other variable in the model (i.e., Figure 2, solid lines). A total indirect path or effect is the part of the association between a maternal weight trait and SGA that does operate via any other variable in the model (i.e., Figure 2, dashed and then solid lines). A total path or effect is the total association (i.e., direct and total indirect) between a maternal weight trait and SGA. SGA was defined according to a birth-weight-for-gestational-age <10th percentile of the INTERGROWTH-21st standard. The model was applied to a subset of participants with a *z*wt_−3 mo_ ≤0.5 *z* scores in whom there was a protective association of the *z*wt_−3 mo_ with SGA risk (Table 2)_._ The model was adjusted for the season of birth (first 4 sets of Fourier terms), sex (female compared with male), parity (1–3 or 4–12 compared with 0), maternal age (decimal years) and height (centimeters) at booking, and intervention (multiple micronutrients, protein energy, or multiple micronutrients plus protein energy compared with iron and folic acid). SGA, small for gestational age; *z*wt_0 mo_, weight *z* score at conception; *z*wt_−3 mo_, weight *z* score at 3 mo preconception; *z*wt_+3 mo_, weight *z* score at 3 mo postconception; *z*wt_+7 mo_, weight *z* score at 7 mo postconception.

2 With the use of only data on the subset of participants, *z* scores were recalculated internally (i.e., observation − mean ÷ SD) at each time point with individual weights (kilograms) that were estimated from the multilevel model shown in Supplemental Table 1.

3 Mean ± SD in parentheses (all such values).

Secondary analyses with continuous outcomes are presented in **Table 4** (conditional maternal weight traits) and **Supplemental Table 5** (maternal weight traits). Note that associations of conditional *z*wt_+3 mo_ and conditional *z*wt_+7 mo_ with infant head-circumference-for-gestational-age *z* score were linear, whereas those with infant weight and length-for-gestational-age *z*-scores were nonlinear and favored women with higher weight gain (in line with the SGA findings). Also, *z*wt_−3 mo_ was associated with infant weight-for-gestational-age *z* score (β = 0.152; 95% CI: 0.063, 0.242) and head-circumference-for-gestational-age *z* score (β = 0.129; 95% CI: 0.026, 0.233), but the conditional *z*wt_0 mo_ was not strongly related to any outcome.

**TABLE 4:** Associations of birth anthropometric-measure *z* scores with conditional maternal weight measures that were estimated with the use of a single general linear regression model for each outcome^1^

	Adjusted model					
	Weight-for-gestational-age *z* score (*n* = 533)		Length-for-gestational-age *z* score (*n* = 553)		Head-circumference-for-gestational-age *z*s core (*n* = 554)	
Maternal or conditional weight *z* score	*B* (95% CI)	*P*	*B* (95% CI)	*P*	*B* (95% CI)	*P*
*z*wt_−3 mo_^2^	0.152 (0.063, 0.242)	0.001	0.080 (−0.025, 0.185)	0.136	0.129 (0.026, 0.233)	0.014
Conditional *z*wt_0 mo_^2^	−0.031 (−0.111, 0.048)	0.438	−0.064 (−0.158, 0.030)	0.181	−0.036 (−0.133, 0.060)	0.461
Conditional *z*wt_+3 mo_^2^	—	—	—	—	0.173 (0.075, 0.271)	0.001
If ≤−0.5 *z* scores^3^	0.134 (−0.067, 0.336)	0.192	0.107 (−0.130, 0.344)	0.376	—	—
If >−0.5 *z* scores^3^	0.162 (0.041, 0.283)	0.009	0.173 (0.029, 0.316)	0.019	—	—
Conditional *z*wt_+7 mo_^2^	—	—	—	—	0.093 (−0.002, 0.187)	0.056
If ≤−0.5 *z* scores^3^	−0.062 (−0.274, 0.150)	0.566	−0.013 (−0.262, 0.235)	0.915	—	—
If >−0.5 *z* scores^3^	0.264 (0.149, 0.379)	<0.001	0.135 (−0.001, 0.271)	0.052	—	—

1 *z* scores were based on INTERGROWTH-21st birth-size-for-gestational-age standards. Each model was adjusted for the season of birth [binary variable: June to October (rainy/hungry) compared with November to May (dry/harvest)], sex (female compared with male), parity (1–3 or 4–12 compared with 0), maternal age (decimal years) and height (centimeters) at booking, and intervention (multiple micronutrients, protein energy, or multiple micronutrients plus protein energy compared with iron and folic acid). Maternal weight *z* scores were calculated internally (i.e., observation − mean ÷ SD) at each time point with the use of the individual weights (kilograms) that were estimated from the multilevel model shown in Supplemental Table 1. Conditional *z* scores were computed as the standardized residuals from regressing weight at one time point on weight at all previous time points with the use of the individual weights (kilograms) that were estimated from the multilevel model shown in Supplemental Table 1; these variables represent the change from the previous time point independent of all previous weights and regression to the mean. *z*wt_0 mo_, weight *z* score at conception; *z*wt_−3 mo_, weight *z* score at 3 mo preconception; *z*wt_+3 mo_, weight *z* score at 3 mo postconception; *z*wt_+7 mo_, weight *z* score at 7 mo postconception.

2 There was no evidence that the associations of *z*wt_−3 mo_ and Conditional *z*wt_0 mo_ with the outcomes were nonlinear, which is why only one estimate is presented for each exposure-outcome association. Similarly, there was no evidence that the associations of Conditional *z*wt_+3 mo_ and Conditional *z*wt_+7 mo_ with the head-circumference outcome were nonlinear, which is why only one estimate is presented for each of these associations.

3 Shape of the association between each maternal or conditional weight *z*-score variable and each outcome was investigated with the use of restricted cubic splines, and nonlinearity was approximated in the models shown in the table with the use of linear splines.

## DISCUSSION

This study investigated how preconceptional and gestational weight trajectories (summarized by individual-level traits) are associated with SGA risk in a population of rural African women and in a setting with high risk of SGA. The first key finding was that greater weight at 3 mo preconception (and at all other time points) was related to lower SGA risk but only in the more-underweight women who had the highest observed SGA rates. In a path analysis that focused only on this subgroup, only weight at 7 mo of gestation had a direct effect on SGA risk. Although this result suggests that any association of preconceptional weight with SGA risk is likely to operate indirectly via weight during pregnancy, it does not suggest that intervening in pregnancy could offset the impact of poor nutritional status before conception. Instead, preconceptional weight is an important determinant of SGA risk because it is highly correlated with maternal weight throughout pregnancy. The second key finding was that greater weight gain between 3 and 7 mo of gestation was related to lower SGA risk but only in women who surpassed a threshold (∼4.5 kg) which was presumably the point at which the nutritional environment can support both the mother and fetus (44, 45). This result is in agreement with our finding of stronger supplementation effects on fetal growth in mothers who showed the greatest gestational weight gain (46). Finally, we showed no evidence that preconceptional and early gestational weight changes were associated with SGA risk.

Informed by previous research, we expected seasonally driven nutrition- and health-related factors in The Gambia to produce a diverse range of maternal weight trajectories (26–30). For example, Prentice et al. (26) reported mean monthly weight gains in pregnant women of 1500 g in the dry/harvest season but only of 400 g in the rainy/hungry season. The multilevel model that is presented in Supplemental Table 1 extends this knowledge by describing fine-tuned trajectories that differ according to the day of the year. Although seasonality itself has previously been mapped onto SGA risk in The Gambia (35, 36), maternal weight trajectories before and early in pregnancy have not. In a previous prenatal trial of a high-energy groundnut-biscuit supplement from ∼20 wk of gestation, each 1-kg/mo increase in gestational weight gain was associated with a 299-g increase in birth weight (47). In agreement, the present study showed that greater weight gain beyond the first trimester was associated with lower SGA risk, but by considering nonlinearity, we revealed that this protective association was most pronounced in more-underweight women.

Outside of The Gambia, observational studies have reported positive relations of prepregnancy weight with birth weight (11–24), but evidence from intervention trials implicating prepregnancy nutritional status has been more limited (48–50). A systematic review of studies that related prepregnancy and early gestational nutrition to maternal and infant outcomes concluded that few well-designed studies on prepregnancy maternal size have been conducted (25). The most common limitation has been that maternal weight was self-reported (often at a single time point) and, therefore, subject to recall and selection biases (25); the present study does not have the same limitation. Nevertheless, the existing literature has reported relations of a range of prepregnancy nutritional-status indicators (e.g., underweight and vitamin supplement use) with a range of birth outcomes (e.g., head circumference and gestational age) (13, 14, 51–56). In addition to SGA, we investigated continuous birth-size-for-gestational-age outcomes. Greater gestational weight gain at any level was associated with larger offspring head circumference, whereas only greater gestational weight gain above a threshold was associated with birth weight and length. This result may reflect better nutrition (related to greater gestational weight gain) being prioritized for brain growth over weight gain and linear growth.

The strength of the present article is the thorough analysis of maternal weight data that were collected serially before and during pregnancy in relation to SGA risk. More specifically, *1*) a robust multilevel model was developed to produce individual-level maternal weight trajectories (while accounting for and investigating the complex impact of seasonality on the sample-average trajectory) with the use of data from the full sample; *2*) the shapes of the associations of maternal weight traits (summarizing the individual trajectories) with SGA were properly captured with the use of linear splines within an appropriate regression framework (i.e., Poisson with robust-error variance) to obtain estimates as RRs; and *3*) a path analysis was used (in a subsample) to correctly estimate the extent to which maternal weight at different time points influenced SGA risk directly or indirectly via maternal weight at subsequent time points. To our knowledge, no other study has been able to look at preconceptional weight and weight change in such detail. In terms of limitations, no weight measure at the end of pregnancy was available, and thus, we could not capture weight change to the end of the third trimester, and it was not prudent to test for an effect modification (e.g., *z*wt_−3 mo_ multiplied by conditional *z*wt_+7 mo_) because of our sample size and model complexity. In support of this decision, other studies have shown no evidence of an interaction between prepregnancy weight and gestational weight gain on birth weight (11). Gestational weight gain will increasingly reflect the size of the fetus as pregnancy progresses, but we were not able to isolate maternal weight from fetal weight. Therefore, associations of maternal weight (and weight gain) during pregnancy with SGA may in part reflect associations of fetal weight (and weight gain) with SGA. We used a 2-stage modeling approach; therefore, our estimates in the second stage may have been biased with 95% CIs that were too narrow (57). A one-stage approach would have required a simpler multilevel model with less-realistic trajectories, and we decided that this was a bigger compromise. Finally, we acknowledge that the presented associations may have been subject to residual confounding and might not be generalizable to other populations.

With acknowledgment of the limitations of observational data, our results may inform nutritional intervention programs to reduce SGA risk in LMIC settings. First, the results suggest a need to target the most undernourished and vulnerable women, perhaps <3 mo preconception because preconceptional and early gestational weight changes were not strongly related to SGA risk, and to maintain any improvement in nutritional status throughout pregnancy. In agreement, previous prenatal trials in The Gambia have been shown to improve gestational weight gain and birth weight more so in the nutritionally debilitating rainy/hungry season than in the dry/harvest season (47, 58). Second, the trials have suggested that any nutritional intervention may need to be sufficient enough for second- and third-trimester gestational weight gains to surpass a threshold. Because the majority of women with low preconceptional weight showed gestational weight gain above the observed threshold in our sample (i.e., 72% of women with *z*wt_−3 mo_ ≤0.5 had conditional *z*wt_+7 mo_ >−0.5), these 2 recommendations are not mutually exclusive. Finally, maternal obesity and excessive gestational weight gain are known to be related to large-for-gestational-age and various cardiometabolic risk factors (59–63), and thus, any intervention program that promotes gestational weight gain will need to be revisited as LMICs undergo nutritional and epidemiologic transitions.

In conclusion, our results suggest that, in resource-poor settings such as The Gambia, any association of greater preconceptional or gestational weight with lower SGA risk may be most pronounced in more undernourished and vulnerable women. Independent of this proposition, greater second- and third-trimester weight gains beyond a threshold may be protective.

## Footnotes

6 Abbreviations used: ENID, Early Nutrition and Immune Development; LMIC, low- and middle-income country; MMN, multiple micronutrient; PE, protein energy; SGA, small for gestational age; *z*wt_0 mo_, weight *z* score at conception; *z*wt_−3 mo_, weight *z* score at 3 mo preconception; *z*wt_+3 mo_, weight *z* score at 3 mo postconception; *z*wt_+7 mo_, weight *z* score at 7 mo postconception.
